# Study on Electrical and Temperature Characteristics of β-Ga_2_O_3_-Based Diodes Controlled by Varying Anode Work Function

**DOI:** 10.3390/nano14242035

**Published:** 2024-12-18

**Authors:** Yunlong He, Baisong Sheng, Xiaoli Lu, Guran Chen, Peng Liu, Ying Zhou, Xichen Wang, Weiwei Chen, Lei Wang, Jun Yang, Xuefeng Zheng, Xiaohua Ma, Yue Hao

**Affiliations:** 1 State Key Laboratory of Wide Bandgap Semiconductor Devices and Integrated Technology, National Engineering Research Center of Wide Band-Gap Semiconductor, School of Microelectronics, Xidian University, Xi’an 710071, China; ylhe@xidian.edu.cn (Y.H.);; 2 State Key Laboratory of Wide Bandgap Semiconductor Devices and Integrated Technology, National Center of Technology Innovation for Wide-Bandgap Semiconductors of Nanjing Co., Ltd., Nanjing 210016, China; 3 China Academy of Space Technology (Xi’an), Xi’an 710100, China

**Keywords:** β-Ga_2_O_3_, diode, turn-on voltage, temperature characteristic, contact resistance

## Abstract

This study systematically investigates the effects of anode metals (Ti/Au and Ni/Au) with different work functions on the electrical and temperature characteristics of β-Ga_2_O_3_-based Schottky barrier diodes (SBDs), junction barrier Schottky diodes (JBSDs) and P-N diodes (PNDs), utilizing Silvaco TCAD simulation software, device fabrication and comparative analysis. From the perspective of transport characteristics, it is observed that the SBD exhibits a lower turn-on voltage and a higher current density. Notably, the V_on_ of the Ti/Au anode SBD is merely 0.2 V, which is the lowest recorded value in the existing literature. The V_on_ and current trend of two types of PNDs are nearly consistent, confirming that the contact between Ti/Au or Ni/Au and NiO_x_ is ohmic. A theoretical derivation reveals the basic principles of the different contact resistances and current variations. With the combination of SBD and PND, the V_on_, current density, and variation rate of the JBSD lie between those of the SBD and PND. In terms of temperature characteristics, all diodes can work well at 200 °C, with both current density and V_on_ showing a decreasing trend as the temperature increases. Among them, the PND with a Ni/Au anode exhibits the best thermal stability, with reductions in V_on_ and current density of 8.20% and 25.31%, respectively, while the SBD with a Ti/Au anode shows the poorest performance, with reductions of 98.56% and 30.73%. Finally, the reverse breakdown (BV) characteristics of all six devices are tested. The average BV values for the PND with Ti/Au and Ni/Au anodes reach 1575 V and 1550 V, respectively. Moreover, although the V_on_ of the JBSD decreases to 0.24 V, its average BV is approximately 220 V. This work could provide valuable insights for the future application of β-Ga_2_O_3_-based diodes in high-power and low-power consumption systems.

## 1. Introduction

β-Ga_2_O_3_ is a typical semiconductor material with a band gap of 4.9 eV and a critical breakdown electric field of 8 MV/cm [1,2]. Owing to its high breakdown strength and low conduction loss [3], its potential applications in high-power electronic devices and circuits are inestimable. In addition, due to its high compatibility with traditional melt growth methodologies [4], its manufacturing cost is controllable compared to that of GaN and SiC semiconductors. Therefore, research on β-Ga_2_O_3_ materials and devices is of great significance.

At present, research on β-Ga_2_O_3_ power diodes is a hot topic, and it is especially essential to understand how to increase their breakdown voltage and reduce their specific on-resistance, because they are useful to generate better power characteristics so as to achieve a higher Baliga’s figure-of-merit (BFOM) [5]. In fact, there are many ways to optimize the BFOM, such as by using field plates [6,7], trenches [8], ion implantation [9], thermal oxidation [10], heterojunctions [11] and composite terminal structures [12,13,14,15], and these methods have been verified to be effective. It is worth pointing out that Li et al. [14] achieved the highest BFOM value of 15.2 GW/cm^2^ by combining thick and low doped drift layers with effective edge termination.

Although research on β-Ga_2_O_3_ power diodes remains focused on improving the BFOM value, in practical applications, the turn-on voltage (V_on_) of a device is also crucial, as it affects the conduction loss of the device. For β-Ga_2_O_3_, due to its smaller electron affinity of 3.15 eV [16], the theoretical barrier height is higher than that for SiC and GaN. Consequently, the turn-on voltage of β-Ga_2_O_3_-based diodes is usually around 0.9 V [17,18], which limits the potential application of β-Ga_2_O_3_-based diodes in switching power supplies. Therefore, it is necessary to conduct further research to lower V_on_. Several methods for reducing the turn-on voltage have been reported, such as the use of a trench structure [8], a high-κ TiO_2_ interlayer [19], hydrogen treatment [20], N_2_O plasma treatment [21] and so on. In addition, a low work function anode has been proven to be effective in decreasing V_on_ [22]. However, low work function anodes can lead to a decrease in breakdown voltage. Additionally, research on the thermal stability and conduction mechanisms of low work function anode diodes remains insufficient.

In this work, the effect of different anode metals on the V_on_ of Schottky barrier diodes (SBDs) was studied by Silvaco TCAD simulation. The definition of V_on_ that we considered is consistent with that of Ref. [21]. It was confirmed that a smaller work function can effectively reduce a device’s V_on_. Therefore, six types of device structures for SBDs, p-NiO_x_/β-Ga_2_O_3_ diodes (PNDs) and junction barrier Schottky diodes (JBSDs) were designed and fabricated. Based on the experimental results, the effects of two anode metals (Ti/Au and Ni/Au) on the forward conduction, temperature stability, and reverse breakdown characteristics are discussed. Then, conclusions are drawn by a specific comparative analysis.

## 2. Simulation Settings

**Figure 1:**
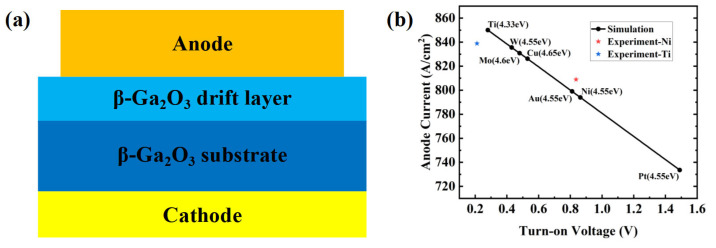
(**a**) Schematics of simulated diode prototype. (**b**) Turn-on voltage and current density curves of anode metal with different work functions.

In conventional diodes, different anode metals have different work functions. A smaller work function can effectively reduce the barrier for electron transitions, which induces the transfer of more electrons to the conduction band. Consequently, under the same forward bias, diodes with lower work function anodes will turn on more easily, resulting in a larger current. Therefore, selecting an anode metal with a low work function is beneficial for reducing the turn-on voltage of a diode. To further verify this, Silvaco TCAD simulation software was used to design a basic diode, as shown in Figure 1a. By selecting different anode metals and considering only the impact of work function on device performance [23], the corresponding turn-on voltage and current density were extracted and are shown in Figure 1b. In this work, the electron affinities of β-Ga_2_O_3_ and NiO_x_ were set to 4.0 eV and 1.8 eV. The bandgap energies of β-Ga_2_O_3_ and NiO_x_ were set to 4.8 eV and 3.7 eV. The dielectric constants of β-Ga_2_O_3_ and NiO_x_ were set to 10.0 and 11.8. The results indicate that when Ti was chosen as the anode metal, the device exhibited a minimum turn-on voltage of 0.3 V and a maximum current density of 850 A/cm^2^. In order to investigate the impact of differences in metal work function on the device characteristics, the self-heating effect, material defects, and variations in metal–semiconductor contact were neglected. Based on these simulation results, β-Ga_2_O_3_-based SBDs, PNDs, and JBSDs with a Ti/Au anode were fabricated and tested. Meanwhile, similar devices with Ni/Au anodes were designed and fabricated for comparison. Due to the lack of p-type Ga_2_O_3_, many other p-type materials were chosen to form heterojunctions. p-type NiO_x_ has been confirmed as an excellent alternative [11,13,14,15]. Therefore, a layer of p-type NiO_x_ was sputtered onto the surface of Ga_2_O_3_ to form the PN junction.

## 3. Experiment and Device Fabrication

The wafer consisted of a 10 μm thick Si-doped β-Ga_2_O_3_ drift layer (with doping concentration of 1.5 × 10^16^ cm^−3^) grown on a conductive (001) β-Ga_2_O_3_ substrate (Sn doping concentration of 2.0 × 10^18^ cm^−3^ and thickness of 640 μm). The fabrication process started with ohmic contacts formed by an alloyed Ti/Au metal stack annealed at 470 °C for 1 min; the wafer was cleaned with HF acid to remove surface impurities and oxides on the anode region, then washed with acetone, ethanol, water and finally dried. Then, the wafer was divided into six regions. For the regions B and E, a p-NiO_x_ layer of about 40 nm was deposited on the whole anode region by sputtering in a mixed gas atmosphere of O_2_/Ar, and for the regions C and F, a p-NiO_x_ layer with the same thickness was selectively deposited on the anode region. The RF power and chamber pressure were set to 100 W and 0.5 Pa in an O_2_/Ar (1:4) mixed ambient. Then, in each region, the metal (Ti/Au or Ni/Au) had to be deposited and then lifted off to form the anode. In this process, a double-layer photoresist with thicknesses of around 0.35 and 2.5 µm was spin-coated. After exposure and development, the photoresist in certain areas was removed. Then, Ti/Au or Ni/Au (45 nm/200 nm) was deposited on the whole anode. After soaking and stripping, all photoresist and metal adhering to it were removed, and the metal was retained in specific areas without the photoresist. In the regions A and D, the metal was in direct contact with the drift layer, which allowed for fabricating the Schottky diodes (SBDs). In the regions B and E, the metal was in contact with NiO_x_, forming a PN junction with Ga_2_O_3_ and thus enabling the fabrication of heterojunction PN diodes (PNDs) in these regions. For the regions C and F, due to the selective deposition of NiO_x_, the metal was in contact with both NiO_x_ and Ga_2_O_3_, allowing for fabricating the junction barrier diodes (JBSDs). To ensure that the comparison was valid, all diodes were manufactured on the same substrate and isolated from each other. The anode metals and diode structures for the six types of samples are listed in Table 1.

**Figure 2:**
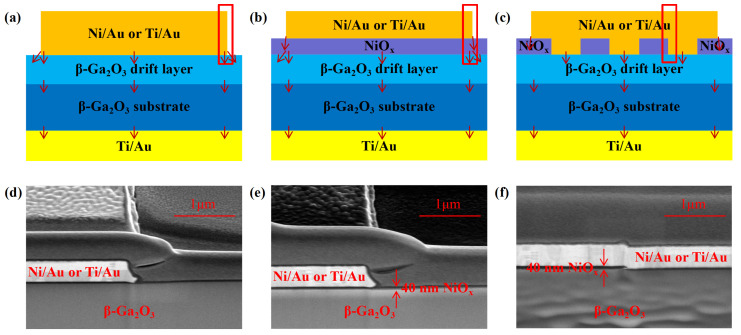
(**a**) Schematics of samples A and D. (**b**) Schematics of samples B and E. (**c**) Schematics of samples C and F. (**d**) Cross-sectional SEM image of samples A and D. (**e**) Cross-sectional SEM image of samples B and E. (**f**) Cross-sectional SEM image of samples C and F.

Circular anode structures were set in all devices; the radius of the anode was 50 μm, and the size of the devices was 60 μm. For the JBSDs, the diameter of a single NiO_x_ structure was 4 µm, and the spacing between adjacent NiO_x_ structures was 5 µm. The structural diagrams and scanning electron microscope (SEM) images of these six devices are shown in Figure 2. The thickness of NiO_x_ was about 40 nm, as shown in Figure 2e,f. The main charge traveling paths are marked in the structure diagrams by red arrows. The Agilent B1500 and B1505 semiconductor parameter analyzers (Agilent, Santa Clara, CA, USA) were used for the measurements.

## 4. Results and Discussion

**Figure 3:**
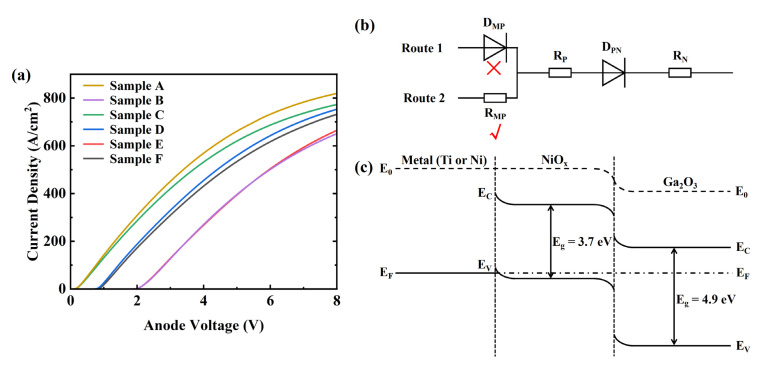
(**a**) Forward I–V characteristics of the six types of devices examined. (**b**) Possible conduction mechanisms for the metal-NiO_x_-Ga_2_O_3_ system. (**c**) Schematic energy band diagram of PNDs.

The forward I–V characteristics of these samples are shown in Figure 3a. The on-resistance (R_on,sp_) was calculated and extracted according to Ref. [21], obtaining values of 0.48, 2.74, 0.58, 1.13, 2.73 and 1.21 mΩ·cm^2^ for the six samples. The results indicated that the turn-on voltages of samples A and D were approximately 0.21 V and 0.81 V, respectively. This is because the work function of Ti is relatively low, at 4.33 eV, significantly reducing the barrier height between the metal and the β-Ga_2_O_3_ drift layer. The V_on_ values for samples B and E were 2.08 V and 2.07 V, respectively. When the applied voltage on the anode was below 6 V, the current curves for samples B and E almost coincided, indicating that Ti/Au and Ni/Au formed the same type of metal-semiconductor contact with p-NiO_x_. Different Schottky metals form different barrier heights, resulting in variations in their current curves. This suggests that the metal/NiO_x_ contact can only be a different form of ohmic contact. The possible conduction mechanisms for the metal-NiO_x_-Ga_2_O_3_ combination are shown in Figure 3b. The contact between metal and NiO_x_ acted as a resistance (R_MP_) for the system. Theoretically, the metal work functions ($W_{m}$) of Ti and Ni are 4.33 eV and 5.15 eV, respectively, while the work function of NiO_x_ is approximately the algebraic sum of its electron affinity ($q\chi_{{NiO}_{x}}$) and bandgap ($E_{g}$). The energy band diagram of PNDs is shown in Figure 3c. Due to the high doping concentration and large work function of p-NiO_x_, it is easy to achieve a good ohmic contact. The holes can be easily injected with a metal (Ti/Au or Ni/Au) and NiO_x_. When the forward voltage is applied, the holes, across the NiO_x_ layer (R_P_), PN junction (D_PN_) and Ga_2_O_3_ layer (R_N_), will allow for the generation of current. Additionally, the PNDs had the highest V_on_, while the SBDs had the lowest V_on_, indicating that the depletion effect of the heterojunction between p-NiO_x_ and β-Ga_2_O_3_ was stronger than that of the Schottky junction between the anode metal and β-Ga_2_O_3_. For the JBSDs, V_on_ was determined by the smaller value between those of the SBDs and PNDs, with the corresponding values for samples C and F being 0.24 V and 0.89 V, respectively. At the same forward voltage, the current density of the JBSDs was slightly lower than that of the SBDs due to the larger depletion region created by the p-NiO_x_/β-Ga_2_O_3_ heterojunction. Despite the metal/NiO_x_ contact types of the two PNDs being consistent, the current density of sample E was slightly higher than that of sample B when the applied voltage on the anode exceeded 6 V, possibly due to different contact resistances between the anode metals and p-NiO_x_. Relevant studies indicated that the contact resistance ($\rho_{c}$) between a metal and a p-type semiconductor is almost unaffected by temperatures around 200 °C [24,25].

Additionally, $\rho_{c}$ satisfies the constraint relationship: $\rho_{c}\propto exp(2\sqrt{\varepsilon_{s}m^{*}}\phi_{B0}/\hbar \sqrt{N})$, where $\varepsilon_{s}$ is the dielectric constant of p-NiO_x_, $m^{*}$ is the effective mass of the carriers, $\phi_{B0}$ is the barrier height, ℏ is the reduced Planck constant, and N is the carrier concentration of p-NiO_x_ [25]. The only difference is that samples B and E had different $\phi_{B0}$ between the anode metal and p-NiO_x_, which could be calculated using the following formula $q\phi_{B0}=E_{g}-q(W_{m}-\chi_{{NiO}_{x}})$[26]. The results indicated that sample E had a lower $\phi_{B0}$, resulting in a smaller $\rho_{c}$. As the applied voltage on the anode gradually increased, the depletion region width decreased. Therefore, the resistance of the PN junction decreased, resulting in the contact resistance between the anode metal and NiO_x_ being more prominent. Consequently, the current density of sample E was slightly higher than that of sample B, and when the applied voltage on the anode reached 8 V, the corresponding current densities of samples B and E were 651 A/cm^2^ and 664.9 A/cm^2^, respectively.

**Figure 4:**
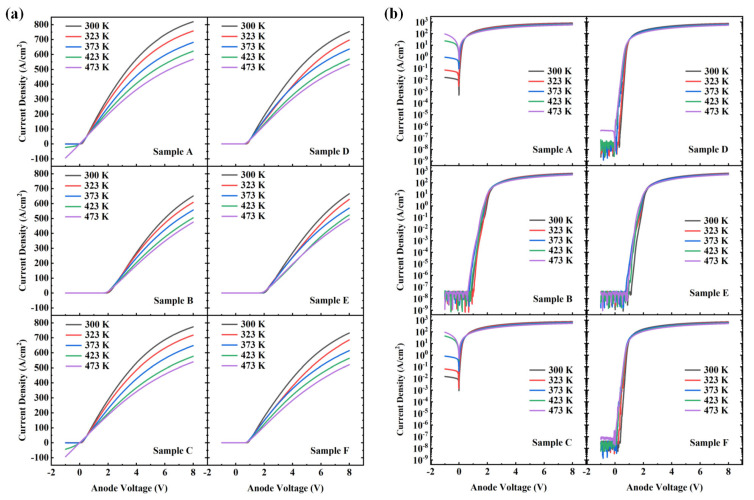
(**a**) Temperature-dependent IV characteristics of the six types of devices in linear plots. (**b**) Temperature-dependent IV characteristics of the six types of devices in semi-logarithmic plots.

**Figure 5:**
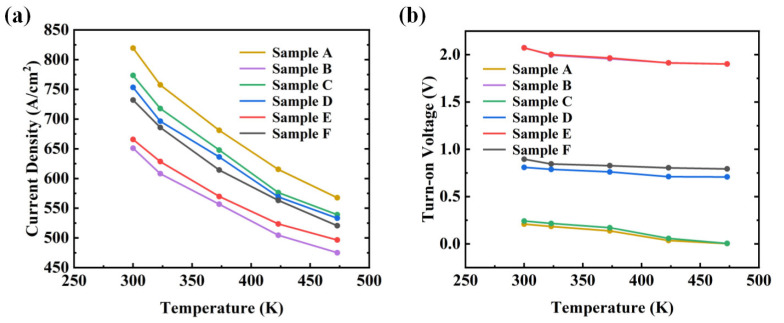
(**a**) Current density curves of the six types of devices. (**b**) Turn-on voltage curves of the six types of devices.

The temperature-dependent I–V curves of these samples are shown in Figure 4 and Figure 5. Figure 4a,b present the I–V curves of the devices under linear and semi-logarithmic scales, respectively. Figure 5a,b provides the variation in current density (at 8 V) and V_on_ as the temperature increased for the six types of samples. For each type of sample, at least three devices were tested, and they provided similar results. Considering the uniformity and consistency of the devices, only a subset of curves and data is presented. According to the thermionic emission (TE) theory [27,28], when a device current approaches saturation, the current density is negatively correlated with temperature. Clearly, all samples exhibited this characteristic. The results indicated that sample A had the highest current density, which is attributed to the presence of only a Schottky junction and a low work function metal. Due to the bandgap narrowing effect [29] and thermally enhanced carrier diffusion [30], the V_on_ of all samples decreased to varying degrees as the external temperature increased. The V_on_ of the two PNDs was almost identical, but the current density at 8 V differed, consistent with previous results. Additionally, for the same anode metal, the turn-on voltage of the JBSDs was slightly higher than that of the SBDs. When the external temperature was set to 473 K, the V_on_ of samples A and C approached zero.

Moreover, Table 2 presents the current density, turn-on voltage, and their respective change rates for the six types of samples at 300 K and 473 K. The data show that sample E had the best thermal stability. Samples D and F exhibited smaller change rates for both V_on_ and current density than samples B and F, which was associated with a higher barrier height. Specifically, the larger work function of Ni increased the barrier for electron transfer from β-Ga_2_O_3_ to the anode metal, thereby maintaining a better thermal stability over a wider temperature range (as described by the thermal electron theory). Similarly, samples B and E had nearly identical V_on_ values at 473 K. However, due to the poorer adaptability of Ti to high-temperature environments, the thermal stability of sample B was slightly lower than that of sample E. In summary, when comparing the three types of devices, the PND exhibited superior thermal stability compared to the SBD, with the JBSD lying in between. Sample E showed the lowest change rates, while sample A had the highest ones, with change rates in current density of 25.31% and 30.73%, respectively. In terms of the variation in V_on_, samples A and C exhibited the largest change rates, indicating that the barrier height between Ti and Ga_2_O_3_ was lower than that between Ni and Ga_2_O_3_, resulting in the poorest thermal stability.

**Figure 6:**
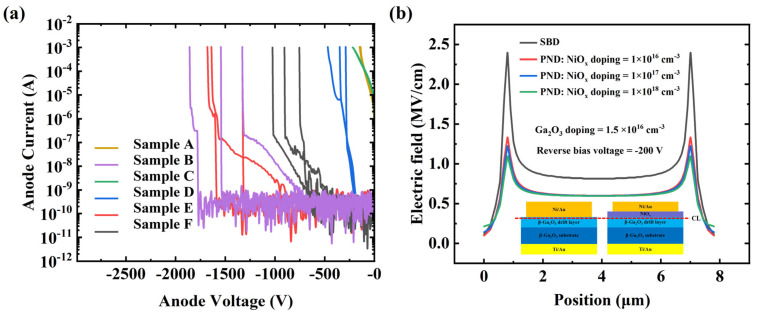
(**a**) The reverse breakdown characteristics of the six types of devices examined. (**b**) The electric field of SBDs and PNDs with different doping concentrations.

The reverse breakdown characteristics of these samples are shown in Figure 6a. In this work, the corresponding reverse anode voltage when the anode current reaches 1 mA is defined as the breakdown voltage (BV). We tested three devices for each of the six types of samples, obtaining average breakdown voltages values of 145, 1575, 220, 370, 1550 and 895 V, respectively. The corresponding values of BFOM (BV^2^/R_on,sp_) were 43.8, 905.3, 83.4, 121.2, 880.0 and 662.0 MW/cm^2^, respectively. Due to the optimal reverse voltage withstand capability of the PN junction, samples B and E had the highest breakdown voltages [31]. Additionally, their BV values were almost identical, further indicating a consistent contact between the anode metal and p-NiO_x_. To confirm that the high BV of the PNDs was primarily due to the voltage tolerance ability of PN junction, a simulation is conducted by Silvaco TCAD. The electric filed of the cut line (CL) was abstracted and is showed in Figure 6b. The result indicates that the PND with a concentration of NiO_x_ of 1 × 10^18^ cm^−3^ had the best electric field dispersion and voltage endurance capability. And owing to the bidirectional depletion effect of the PN junction, the PNDs exhibited a lower peak electric field than the SBDs. Furthermore, the results show that the BV of sample D was higher than that of sample A. This is because Ti has a smaller work function, which reduces the barrier for carrier transition, thereby increasing the probability of electron tunneling in the off-state and resulting in a higher reverse leakage current [32]. Moreover, it was found that the V_on_ of sample C was approximately 0.24 V, while its breakdown voltage could reach 220 V without any field terminal structure. This indicates that β-Ga_2_O_3_-based diodes can achieve a low power consumption and a high breakdown voltage.

## 5. Conclusions

In conclusion, this work systematically investigated the effects of anode metals with different work functions (Ti/Au and Ni/Au) on the electrical characteristics and temperature behavior of β-Ga_2_O_3_-based SBDs, JBSDs, and PNDs, utilizing Silvaco TCAD simulation software for a mechanistic analysis. To this end, six types of diodes with Ti/Au and Ni/Au were designed and fabricated. Through testing and analysis, it was observed that the SBD exhibited a lower turn-on voltage and a higher current density. Notably, the V_on_ of the Ti/Au-anode SBD was merely 0.2 V, which is the lowest recorded value in the existing literature. Furthermore, our study revealed that the turn-on voltages of the two types of PNDs were nearly identical, confirming that the contact between Ti/Au or Ni/Au and NiO_x_ is ohmic, and the contact between Ni/Au and NiO_x_ exhibits a lower contact resistance. The JBSD combines the structures and characteristics of SBD and PND. The V_on_, current density, and variation rate of the JBSD lay between those of the SBD and the PND. In terms of temperature characteristics, all diodes appeared to work well at 200 °C, with both current density and V_on_ showing a decreasing trend as the temperature increased. Among them, the PND with a Ni/Au anode exhibited the best thermal stability, with reductions in V_on_ and current density of 8.20% and 25.31%, respectively, while the SBD with a Ti/Au anode showed the poorest performance, with reductions of 98.56% and 30.73%. Finally, the BV characteristics of all six devices were tested and analyzed. The average BV for the PNDs with Ti/Au and Ni/Au anodes reached 1575 V and 1550 V, respectively. Moreover, although the V_on_ of the JBSD decreased to 0.24 V, its average BV reached approximately 220 V. Every type of sample has its own advantages and disadvantages for high-power applications. Specifically, the SBD has a lower turn-on voltage, higher current density and faster switching. The PND has a higher breakdown voltage and better thermal stability. The JBSD combines the structures and characteristics of SBD and PND. This work aimed to provide valuable insights for the future development of power diodes that are compatible with both low turn-on voltage and high breakdown voltage. We hope that this work can provide valuable insights for the future application of β-Ga_2_O_3_-based diodes in high-power and low-power consumption systems.

## Figures and Tables

**Table 1:** The structures and anode metals of the six types of samples prepared in this study.

Samples	A	B	C	D	E	F
Structure	SBD	PND	JBSD	SBD	PND	JBSD
Anode metal	Ti/Au	Ti/Au	Ti/Au	Ni/Au	Ni/Au	Ni/Au

**Table 2:** The data of current density and turn-on voltage at 300 K and 473 K.

Samples	A	B	C	D	E	F
V_on_ at 300 K (V)	0.209	2.072	0.242	0.809	2.072	0.895
V_on_ at 473 K (V)	0.003	1.901	0.005	0.708	1.902	0.793
Variation value of V_on_ (V)	0.206	0.171	0.237	0.101	0.170	0.102
Variation rate of V_on_	98.56%	8.25%	97.93%	12.48%	8.20%	11.40%
Current density at 300 K (A/cm^2^)	819.4	651.0	770.9	753.4	664.9	732.1
Current density at 473 K (A/cm^2^)	567.6	475.2	541.6	533.3	496.6	520.5
Variation value of current density (A/cm^2^)	251.8	175.8	229.3	220.1	168.3	211.6
Variation rate of current density	30.73%	27.00%	30.29%	29.21%	25.31%	28.90%

## Data Availability

The original contributions presented in the study are included in the article, further inquiries can be directed to the corresponding author/s.
